# Neuroinflammatory astrocytes generated from cord blood-derived human induced pluripotent stem cells

**DOI:** 10.1186/s12974-019-1553-x

**Published:** 2019-08-09

**Authors:** Qiong Zhou, Coralie Viollet, Anastasia Efthymiou, Guzal Khayrullina, Kasey E. Moritz, Matthew D. Wilkerson, Gauthaman Sukumar, Clifton L. Dalgard, Martin L. Doughty

**Affiliations:** 1 Center for Neuroscience and Regenerative Medicine, Uniformed Services University of the Health Sciences, Bethesda, MD 20814 USA; 2 Boehringer Ingelheim Pharma GmbH & Co. KG, Computational Biology, Biberach, Germany; 3 Ronald M. Loeb Center for Alzheimer’s Disease, Icahn School of Medicine at Mount Sinai, New York, NY 10029 USA; 4 Department of Anatomy, Physiology and Genetics, Center for Neuroscience and Regenerative Medicine, Uniformed Services University of the Health Sciences, Bethesda, MD 20814 USA; 5 Viral Immunology and Intravital Imaging Section, National Institute of Neurological Disorders and Stroke, National Institutes of Health, Bethesda, MD 20892 USA; 6 Collaborative Health Initiative Research Program, Uniformed Services University of the Health Sciences, Bethesda, MD 20814 USA

**Keywords:** Human induced pluripotent stem cell, Astrocyte, RNA sequencing, Glutamate uptake, Phagocytosis

## Abstract

**Background:**

Astrocytes respond to central nervous system (CNS) injury and disease by transforming to a reactive astrogliosis cell state that can contribute to either CNS dysfunction or repair. Neuroinflammation is a powerful driver of a harmful A1 astrogliosis phenotype associated with in vitro neurotoxicity and histopathology in human neurodegenerative diseases. Here we report a protocol for the rapid development of a human cell culture model of neuroinflammatory astrogliosis using induced pluripotent stem cells (iPSCs).

**Methods:**

Using RNA sequencing and in vitro cell assays, we measured transcriptional and cellular effects of chronic exposure of human iPSC-derived astrocytes to the cytokines TNFα (tumor necrosis factor alpha) or IL-1β (interleukin-1 beta).

**Results:**

We show TNFα and IL-1β induce pro-inflammatory gene signatures but by widely different magnitudes. TNFα treatment results in 606 differential expressed genes, the suppression of glutamate-uptake, and increased phagocytic activity in astrocyte cultures. In contrast, IL-1β effects are attenuated to 33 differential expressed genes and no significant effects on glutamate-uptake or increased phagocytic activity.

**Conclusion:**

Our approach demonstrates a rapid tool for modeling neuroinflammatory human astrocytic responses in nervous system trauma and disease. In particular, we reveal a model for robust TNFα-induced human astrogliosis suitable for the study of neurotoxic A1 astrocytes.

## Highlights

Astrocytes rapidly differentiated from neural stem progenitor cells (NSPCs) of human cord blood-derived iPSC origin are differentially sensitive to neuroinflammatory cytokines. Chronic TNFα treatment results in widespread differential gene expression, suppressed glutamate uptake, and increased phagocytic activity in cultured iPSC-derived astrocytes. In contrast, IL-1β effects are attenuated with few differentially expressed genes and no effect on glutamate uptake or phagocytic activity.

## Background

Astrocytes are abundant cells in the CNS contributing ~ 40% of all cells in the human brain [1]. Astrocytes function in a range of neurodevelopmental and homeostatic processes in the brain that include the establishment and maintenance of synapses [2]; the regulation of energy supply [3], of extracellular neurotransmitter levels [4], and of brain blood flow [5]; and the maintenance of the blood-brain barrier [6].

Consistent with their wide-ranging homeostatic role, astrocyte dysfunction is linked to a number of neurodevelopmental disorders such as autism spectrum disorder [7] and Down’s syndrome [8] and neurodegenerative diseases such as Alzheimer’s [9] and Parkinson’s disease [10]. Astrocytes are acutely sensitive to changes in their cellular environment and react to CNS damage and disease with changes in gene expression, morphology, and function. This reactive astrocyte state is referred to as astrogliosis [11].

Astrogliosis is a heterogeneous, stimulus-dependent cell state. Middle cerebral artery (MCA) occlusion or lipopolysaccharide (LPS) induced neuroinflammation drive transcriptionally distinct astroglia [12] characterized by divergent neuroprotective or neurotoxic phenotypes respectively [13]. Neurotoxic astroglia, also referred to as A1 astrocytes, are induced by LPS-activated microglia through microglial secretion of the neuroinflammatory cytokines TNFα, IL-1α, and the complement component C1q [13]. LPS also induces increased IL-1β secretion by neuroinflammatory microglia, but unlike TNFα, IL-1α, or C1q, IL-1β treatment alone does not induce astrocyte expression of A1 associated transcripts. These data reveal differential sensitivity to the classical neuroinflammatory cytokines TNFα and IL-1β in astrocytes.

Although rodent animal and cell culture models have provided important data on astrocyte neuroinflammatory signaling, increasing evidence suggests human astrocytes are morphologically, transcriptionally, and functionally distinct from those of rodents. Human astrocytes are morphologically larger and more complex than their rodent counterparts [14], and RNA sequencing of acutely isolated astrocytes reveals a gene expression signature unique to human astrocytes [1]. Xenotransplantation of human astrocytes into the brains of developing mice enhances behavioral learning and long-term potentiation (LTP) in the hippocampus with engrafted human astrocytes exhibiting accelerated Ca^2+^ wave propagation compared to host astrocytes [15]. Furthermore, human astrocytes retain their complex morphology and distinct Ca^2+^ responses when stimulated in vitro [1]. Combined, these data highlight the importance and feasibility of adopting human astrocyte model culture systems to investigate astrocyte pathology in brain trauma and disease.

Induced pluripotent stem cell (iPSC)-derived astrocytes offer an increasingly popular method for modeling physiological and pathological human astrocyte functions in culture [8, 16–21]. Although this approach remains hampered by gaps in our understanding of the signaling events that drive topographical and functional subtype specificity in astrocytes, human iPSC-derived astrocytes have revealed novel pathological features in neurodevelopmental disorders [8] and neurodegenerative disease [21]. In this study, we characterize the neuroinflammatory responses of astrocytes rapidly derived from neural stem progenitor cells (NSPCs) from the human iPSC line NCRM-1. NCRM-1 iPSCs were generated from CD34+ human cord blood cells by episomal plasmid reprogramming [22] and differentiated to positional naïve NSPCs through an embryoid body (EB) method [23]. NSPCs were differentiated to astrocytes as previously described [16, 17] and treated with pro-inflammatory cytokines TNFα or IL-1β. Expression profiling and in vitro glutamate uptake and phagocytosis assays reveal a robust astrocyte inflammatory response to TNFα. In contrast, IL-1β effects are attenuated. We conclude our approach reveals a rapid and efficient protocol for modeling A1-like TNFα inflammatory responses in human astrocytes in CNS disease or trauma.

## Methods

### Human iPSC-derived NSPC cell culture and astrocyte differentiation

In this study, we use neural stem progenitor cells (NSPCs) derived from the human induced pluripotent stem cell (iPSC) line NCRM-1 [24, 25]. NCRM-1 iPSCs were generated in collaboration with the NIH, Sigma-Aldrich (now MilliporeSigma), and XCell Sciences from CD 34+ human cord blood cells by episomal plasmid reprogramming https://www.nimhgenetics.org/stem_cells/crm_lines.php. NCRM-1 iPSCs were differentiated to NSPCs through an embryoid body (EB) method by XCell Sciences. NSPCs were maintained as previously described [24] in Neural Stem Cell Medium (NSCM) on Geltrex® (Life Technologies) coated plates. NSCM consisted of Neurobasal Medium, GlutaMAX™, non-essential amino acids, B27 supplement (all from Life Technologies, Carlsbad, CA), and 10 ng/mL fibroblast growth factor-2 (FGF2) (R&D Systems, Minneapolis, MN). Media was refreshed every other day, and cells were passaged at a 1:3 ratio once the cells reached ~ 80% confluence.

NCRM-1 NSPCs were differentiated to astrocytes as previously described [16]. Briefly, NSPCs were passaged to 1 × 10^6^ cells per well of a six-well plate. Media was changed to Astrocyte Differentiation Medium (ADM) after 24 h. ADM contained DMEM/F12 supplemented with GlutaMAX™, 1.8% BSA, 1x StemPro hESC Supplement (all from Life Technologies), 10 ng/mL ActivinA (R&D Systems), 20 ng/mL Heregulin1b (R&D Systems), 10 ng/mL IGF-1 (PeproTech, Rocky Hill, NJ), and 10 ng/mL FGF2 (R&D) supplemented with 1% fetal bovine serum. Cells were passaged every 4 days at a 1:3 ratio. Cells used for imaging were passaged to Geltrex-coated coverslips 2 days before immunocytochemistry. Astrocyte fate was confirmed when ≈ 100% of the cells were CD44+ and ≈ 50% of the cells were GFAP+ (between 35 and 70 days in ADM).

### Cytokine treatment

Drug treatment on NCRM-1 astrocytes was performed on cultures containing ~ 100% CD44+ cells and ≥ 50% GFAP+ cells. Astrocytes were derived as described previously and plated onto Geltrex-coated 96-well plates after 35–50 days culture in astrocyte differentiation conditions. Astrocytes were treated with 10, 30, or 100 ng/mL of tumor necrosis factor alpha (TNFα, PeproTech, Rocky Hill, NJ) or with 10, 30, or 100 μg/mL of interleukin-1 beta (IL-1β, PeproTech) for 7 days. Controls were treated with PBS/BSA vehicle. The media was replaced with fresh media + cytokine/vehicle after 4 days of treatment.

### Western blot

Whole-cell protein extracts were obtained from astrocyte cultures using NE-PER® reagents (Thermo Scientific) according to the manufacturer’s instructions. Protein fractions were stored at − 20 °C in 1x Halt™/1x Halt™ Phosphatase Inhibitor Cocktail (both Thermo Scientific) until use. Twenty micrograms of protein was loaded per lane and samples separated by SDS-PAGE and electro-transferred to PVDF membrane. For TNF-R1 and IL-1R1 blots, human PBMC (gift of A. Snow) and human MOLT-4 (Abcam, Cambridge, MA) cell lysate samples were included as positive controls respectively. Washes and antisera incubations were performed in 5% skim milk or 5% BSA in TBS with 0.1% Tween-20. Blots were incubated with primary antisera overnight at 4 °C on a rocking platform. Primary antisera and dilutions used were as follows: 1:1000 rabbit polyclonal anti-NF-κβ p65 (Cell Sig. Tech., Danvers, MA); 1:1000 rabbit phospho-anti-NF-κβ p65 (Ser536) (Cell Sig. Tech); 1:1000 rabbit polyclonal anti-IL-1R1 (Abcam); 1:1000 rabbit polyclonal anti-TNF-R1 (Cell Sig. Tech); 1:1000 rabbit anti-Actb (Cell Sig. Tech); and 1:1000 mouse monoclonal anti-Gapdh (MilliporeSigma, St. Louis, MO). The following day, blots were washed 3 times and incubated with a 1:5000 dilution of peroxidase-conjugated goat anti-mouse or anti-rabbit IgG secondary antisera (Cell Sig. Tech.) for 1 h at room temperature. Blots were developed in Immobilon chemiluminescent ECL substrate (Millipore) for 5 min at room temperature and the fluorescent signal captured using a BIO-RAD ChemiDoc Touch imaging system (BIO-RAD, Hercules, CA 94547). Western blot data was generated from three experimental replicates and immunosignal intensities (protein density) measured using BIO-RAD software. For each replicate, phospho-anti-NF-κβ immunosignals were normalized to anti-NF-κβ immunosignals and the densitometry data was presented as the normalized mean signal ± SD.

### Transcriptome profiling by RNA sequencing

Total RNA from harvested iPSC culture was quantified by fluorescence dye-based methodology (RiboGreen) on a Spectramax Gemini XPS plate reader (Molecular Devices, Mountain View, CA). RNA integrity was assessed using automated gel-based electrophoresis on an Experion automated electrophoresis system (Bio-Rad, Hercules, CA). Samples used as input for library preparation were RNA integrity number (RIN) value > 8. Total RNA input amount of 200 ng was used for library preparation using the TruSeq Stranded mRNA Library Preparation Kit (Illumina, San Diego, CA). Sequencing library concentration was measured by quantitative PCR using KAPA Library Quantification Kit for NGS (Kapa, Wilmington, MA) on a CFX384 real-time PCR detection system (Bio-Rad, Hercules, CA) and assessed for size distribution on an Experion automated electrophoresis system (Bio-Rad). Sequencing libraries were pooled and sequenced on a NextSeq 500 desktop sequencer (Illumina) using a NextSeq 500 High Output v2 Kit with paired-end reads at 75 bp length. Raw sequencing data was demultiplexed using bcl2fastq2 Conversion Software 2.17 before alignment using TopHat Alignment v1.0 and differential expression analysis using Cufflinks Assembly & DE v1.1.0 on BaseSpace Onsite (Illumina) with the Illumina iGenomes UCSC hg19 reference and GTF transcript annotation files. Comparative analysis of transcript abundance values was performed using GenePattern 3.9 [26] with comparative marker selection features analyzed for gene ontology enrichment using PANTHER Classification System [27]. The Database for Annotation, Visualization and Integrated Discovery (DAVID) v6.8 was utilized for ontology and pathway enrichment analysis [28]. RNA sequencing was performed on a minimum three biological replicates for each treatment group.

### Real-time quantitative reverse transcription PCR (qRT-PCR)

First-strand cDNA was synthesized from 1 μg of total RNA using random primer/oligo (dT) primer according to the manufacturer’s instructions (ABI, Carlsbad, CA). Synthesized cDNA was diluted to final concentration of 10 ng/μl for qPCR using SYBR Green Master Mix (ABI) on a Thermal cycler C1000 with CFX384 or CFX96 real-time system. Optimum primers were designed using NCBI’s Primer BLAST www.ncbi.nlm.nih.gov/tools/primer-blast/. Primer sequence pairs used are detailed in Table 1. Primer specificity was confirmed by verifying a single PCR product had been generated using UV gel electrophoresis, as well as by confirming the melting temperature of the product had a single value on dissociation plots. Relative expression levels were calculated using the Bio-Rad CFX Manager 3.1 software. Briefly, gene expression was normalized (∆Cq) for each gene to the highest expression level by dividing the expression level of each sample by the highest level of expression in all the samples. The software sets the highest level of expression to a value of 1 and re-scales all the sample expression levels. The expression was then normalized (∆∆Cq) to the quantities of reference house-keeping (HK) genes ACTB and GAPDH to give a relative expression level.

**Table 1:** Primer sequences used for real-time quantitative reverse transcription PCR (qRT-PCR)

Gene	Forward primer	Reverse primer
C3	TAC AAC GTG GAG GCC ACA TC	ACG GGA GGC ACA AAG TCA AA
CCL2	CAG CCA CCT TCA TTC CCC AA	GAC ACT TGC TGC TGG TGA TTC
CXCL10	TGA ATC CAG AAT CGA AGG CCA	TGC ATC GAT TTT GCT CCC CT
CXCL11	AAG CAG TGA AAG TGG CAG AT	AAG CCT TGC TTG CTT CGA T
IL-1R1	GGA GGA CTT GTG TGC CCT TA	CCA CAT TCA TCA CGA TGA GCC
IL-8	AAG GTG CAG TTT TGC CAA GG	GTG TGG TCC ACT CTC AAT CAC T
MMP9	GTA CTC GAC CTG TAC CAG CG	AGA AGC CCC ACT TCT TGT CG
TNFSR1	TCC TGT AGT AAC TGT AAG AAA AGC C	AGA AAA TGA CCA GGG GCA ACA

### Immunocytochemistry

Immunocytochemistry was performed as previously described [29] on cells on Geltrex-coated glass coverslips. Cells were washed once with warmed basal medium (DMEM/F12 or Neurobasal Medium) and fixed with 4% paraformaldehyde in DMEM/F12 for 20 min at room temperature or at 4 °C overnight. Fixed cells were then washed three times with PBS and blocked using a buffer containing 10% normal goat serum (NGS) for 1 h at room temperature. Primary antibody was diluted in incubation buffer containing 0.1% Triton X-100 in PBS and 1% NGS, and incubation was performed at 4 °C overnight or 2 h at room temperature. Cells were then washed again with PBS three times, and secondary antibody was diluted in incubation buffer. Secondary antibody incubation was performed for 1 h at room temperature. Cells were then washed twice with PBS and mounted onto slides with Mowiol and allowed to dry at room temperature overnight before imaging. Alexa fluor-conjugated secondary antibodies (Life Technologies) were used for single and double labeling, and all secondary antibodies were tested for specificity.

The primary antibodies used are rat monoclonal anti-CD44 at 1:1000 dilution (ThermoFisher Scientific, Waltham, MA); rabbit polyclonal anti-GFAP antibody at 1:1000 dilution (DAKO, Glostrup, Denmark); mouse monoclonal anti-S100β at 1:1000 dilution (MilliporeSigma); rabbit anti-VIMENTIN at 1: 250 dilution (Cell Sig. Tech., Danvers, MA); and rabbit anti-NF-κβ at 1:1000 dilution (Cell Sig. Tech.). Secondary antibodies used are Alexa Fluor 555 Goat Anti-Mouse and Alexa Fluor 488 Goat Anti-Rabbit (Life Technologies; Carlsbad, CA). DAPI (Molecular Probes, Thermo Fisher) at 1:2000 dilution was used for nuclei staining. Images were captured using Zeiss 700 and 710 Laser Scanning Confocal Microscopes (Zeiss, Thornwood, NY).

### Glutamate uptake assay

1 × 10^5^ NCRM-1 astrocytes were plated per well of a 24-well plate and treated with TNFα or IL-1β as described. To test for glutamate uptake in astrocyte cultures, the media was removed and astrocytes equilibrated in pre-warmed Hanks balanced salt solution (HBSS) for 10 min at 37 °C, 5% CO_2_. Astrocytes were then incubated for 1 h at 37 °C, 5% CO_2_ in HBSS/0.1 mM L-glutamate (Abcam). Fifty microliters of HBSS/L-glutamate was then removed and the glutamate content determined using a Glutamate Assay Kit (Abcam) according to the manufacturer’s instructions. Glutamate uptake data were collected from three biological replicates.

### Phagocytosis assay

Red (Ex580/Em605) carboxylate-modified microspheres (FluoSpheres, F8821, ThermoFisher Scientific) were obtained and prepared as previously described [30]. Briefly, FluoSpheres were pelleted at 10,000*g* for 15 min at room temperature. Supernatant was removed, and the pellet was washed in pure water and pelleted again. Following, water was removed and FluoSpheres were re-suspended (3% BSA, 25 mM Na_2_HPO_4_, pH 6.0) by rapid vortexing.

5 × 10^5^ NCRM-1 astrocytes were plated per well of a six-well plate and cultures treated with 100 μg/ml TNFα, IL-1β, or with PBS/BSA vehicle control for 7 days (the media/cytokine/vehicle was refreshed after 4 days). Fifteen microliters of beads were added directly to each well for 30 min at 37 °C. To collect cells for analysis, media was removed and cells were washed twice with PBS, then gently scraped and centrifuged at 300*g* for 10 min. Cell pellets were re-suspended in FACS buffer and submitted to flow cytometry (BD Accuri C6, BD Biosciences, Franklin Lakes, NJ). Cells were gated so that only viable cells were counted. Ten thousand cells per treatment group were counted. To adjust for background, control cells that did not have beads were used for each experiment. The mean fluorescence of all viable cells was measured, and data were analyzed using unpaired Student’s *t* tests.

### Data analysis and statistics

All data are presented as mean ± standard deviation of the mean unless otherwise stated. All data was using one-way ANOVA with Sidak’s or Tukey’s multiple comparisons test or by unpaired Student’s *t* test (GraphPad Prism). A *p* value of < 0.05 was considered significant.

## Results

### Human NCRM-1 NSPCs can be rapidly differentiated to TNFα sensitive astrocytes

**Fig. 1:**
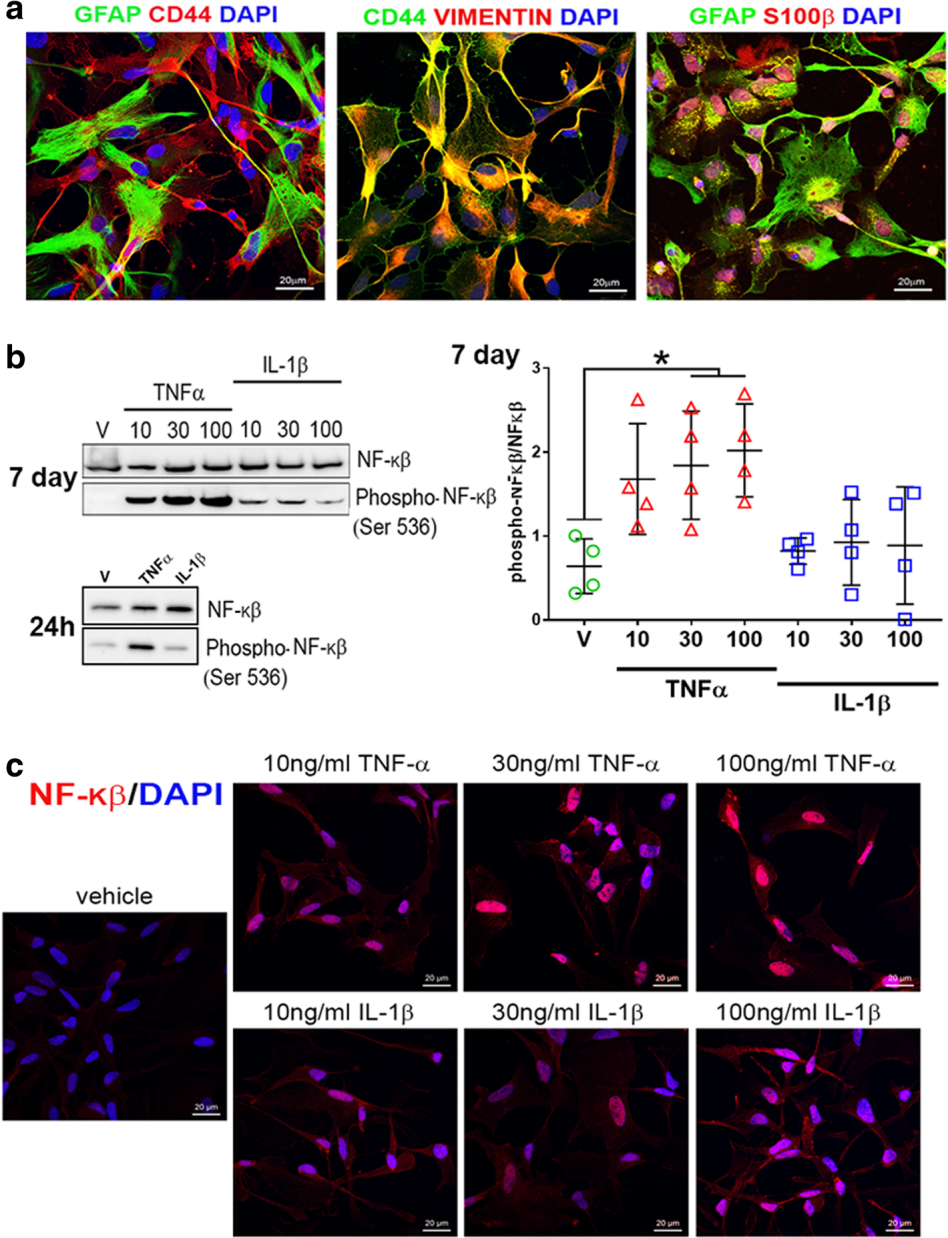
Differentiated astrocytes from human stem cells respond to cytokines. **a** NCRM-1 iPSC-derived NSPCs differentiated to astrocytes for 5–7 weeks in vitro express glial cell lineage markers CD44, GFAP, VIMENTIN, and S100β. **b** Chronic TNFα but not IL-1β treatment results in a significant dose-dependent increase in NF-κβ phosphorylation measured by Western blot densitometry. *p < 0.05 one-way ANOVA with Tukey’s multiple comparisons test. Data are presented as mean ± SD, *n* = 3. **c** Anti-NF-κβ immunocytochemistry reveals increased NF-κβ nuclear localization with chronic TNFα treatment

In order to test the inflammatory responses of human astrocytes, we differentiated astrocytes from NCRM-1 NSPCs as described previously [16, 17]. This protocol results in the rapid differentiation of NSPCs to CD44+/GFAP/S100β+/VIMENTIN+ astrocytes within ~ 35–50 days (Fig. 1a). RNA-seq profiling of astrocytes reveals the upregulation of astrocytic and downregulation of neuronal genes compared to NSPCs and an immature astrocytic gene signature more consistent with human fetal astrocyte progenitors (APCs) than mature astrocytes, see [17].

We next treated NCRM-1 astrocytes with pro-inflammatory cytokines TNFα or IL-1β for 7 days in culture to examine the effects of chronic treatment on cell signaling. Cytokines were added to cell culture media at concentrations of 10, 30, or 100 ng/mL. Western blots demonstrate a significant (*p* < 0.05) increase in phosphorylation of the transcription factor NF-κβ with 30 and 100 ng/mL TNFα treatment (Fig. 1b). Increased NF-κβ phosphorylation is detected at 24 h with 100 ng/mL TNFα treatment, and immunocytochemistry reveals increased NF-κβ nuclear localization with TNFα treatment compared to vehicle controls (Fig. 1c). In contrast, IL-1β-treated phospho-NF-κβ levels were statistically unchanged when compared to vehicle controls (Fig. 1b) and NF-κB nuclear localization noticeably lower when compared to TNFα-treated cultures (Fig. 1c). Elevated phosphorylation and nuclear localization of NF-κβ are consistent with activated NF-κβ signaling in NCRM-1 astrocytes in response to chronic TNFα treatment. In contrast, the data indicate NF-κβ signaling is unaffected or attenuated (to below detection levels) with IL-1β treatment of NCRM-1 astrocytes.

### TNFα suppresses glutamate uptake and enhances phagocytic activity in human NCRM-1 astrocytes

**Fig. 2:**
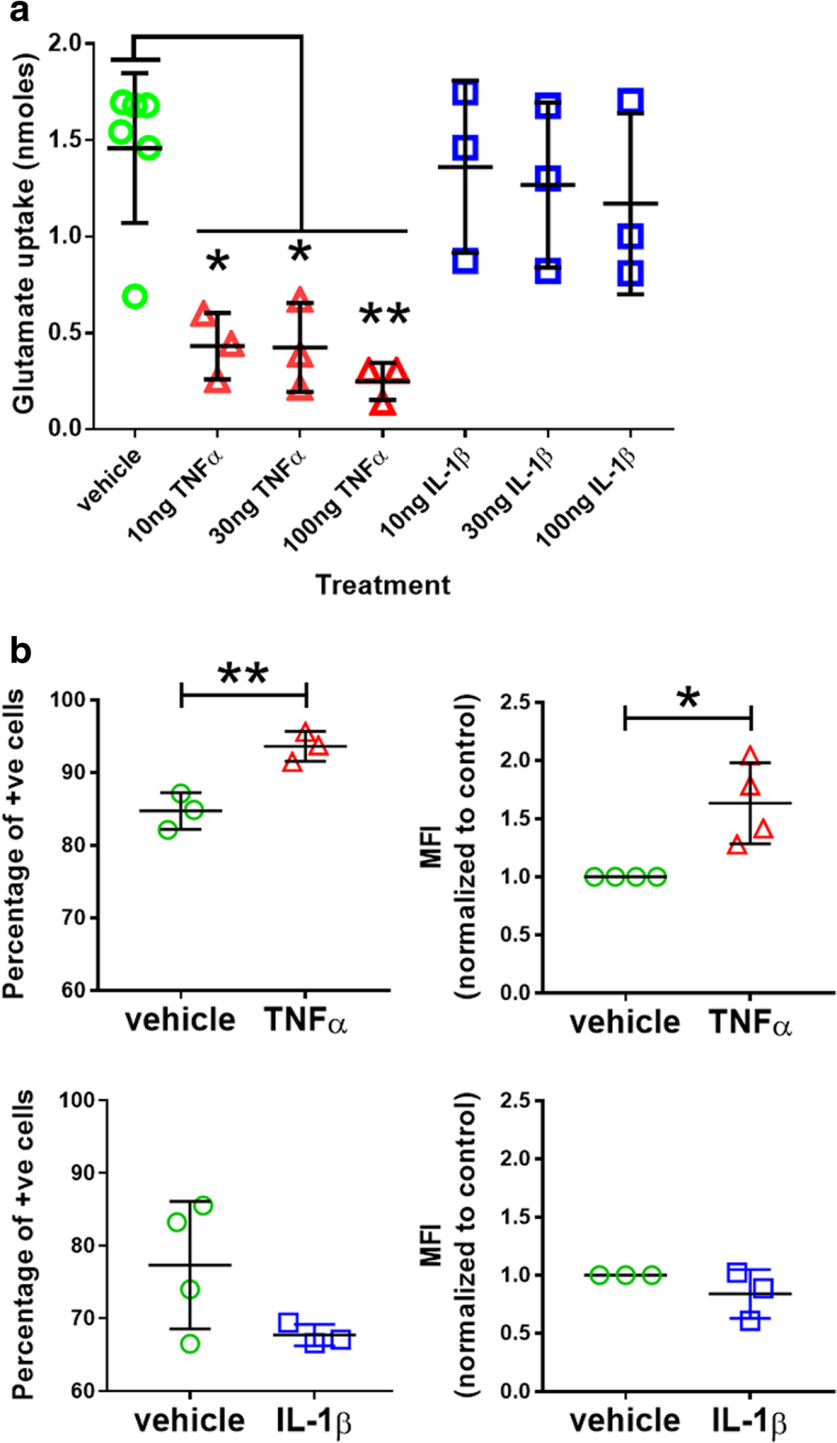
TNFα but not IL-1β treatment suppresses glutamate uptake and enhances phagocytic activity by differentiated astrocytes. **a** Glutamate uptake by astrocyte cultures is significantly reduced following chronic treatment with TNFα but not IL-1β (**p < 0.01, *p < 0.05 one-way ANOVA with Tukey’s multiple comparisons test, cytokine *n* = 3, vehicle *n* = 6). **b** The number of phagocytic astrocytes (measured as the percent of fluorescent positive cells) and the rate of phagocytosis (measured as the mean fluorescent intensity, MFI) are significantly increased by TNFα but not IL-1β. **p < 0.01, *p < 0.05 unpaired Student’s *t* test, *n* = 3–4. Data are presented as mean ± SD

We next examined the effects of chronic TNFα or IL-1β treatment on homeostatic functions of NCRM-1 astrocytes. Astrocytes play a central role in establishing extracellular levels of the excitatory neurotransmitter glutamate in the CNS via active glutamate uptake by excitatory amino acid transporter 1 and 2 (EAAT1 and EAAT2) [31]. We tested the effects of chronic cytokine treatment on glutamate uptake by NCRM-1 astrocytes. Following 7-day cytokine (10, 30 or 100 ng/mL) or vehicle treatment, astrocytes were incubated for 1 h in Hanks balanced salt solution (HBSS) of 0.1 mM L-glutamate. HBSS glutamate levels were then measured by colorimetric assay to quantify the amount of glutamate uptake. The assay reveals a significant (*p* < 0.05 to *p* < 0.01) dose-dependent reduction in glutamate uptake with TNFα treatment (Fig. 2a). In contrast glutamate uptake was not significantly reduced by IL-1β, although mean uptake values were marginally decreased with increasing IL-1β dose indicating a possible attenuated dose-dependent response (Fig. 2a).

Recent evidence has revealed phagocytic activity by astrocytes. Astrocytes actively engulf synapses in the developing and adult brain in neural circuit refinement [32] and engulf cell debris in CNS trauma and disease [33, 34]. We used fluorescent microbeads and flow cytometry to measure phagocytosis by NCRM-1 astrocytes following chronic treatment of 100 ng/mL TNFα or IL-1β compared to vehicle control. The data revealed increased phagocytic activity in TNFα but not IL-1β treated cultures (Fig. 2b). TNFα significantly increased the percentage of phagocytic cells (*p* < 0.01) and the mean fluorescence intensity (MFI) of cells (*p* < 0.05) indicating an increased number of phagocytic astrocytes and increased phagocytic activity of individual astrocytes in response to TNFα. In contrast, IL-1β had no significant effect of the percentage or MFI of NCRM-1 astrocytes compared to vehicle controls.

### Cytokines TNFα and IL-1β both upregulate pro-inflammatory genes but by widely different magnitudes

**Fig. 3:**
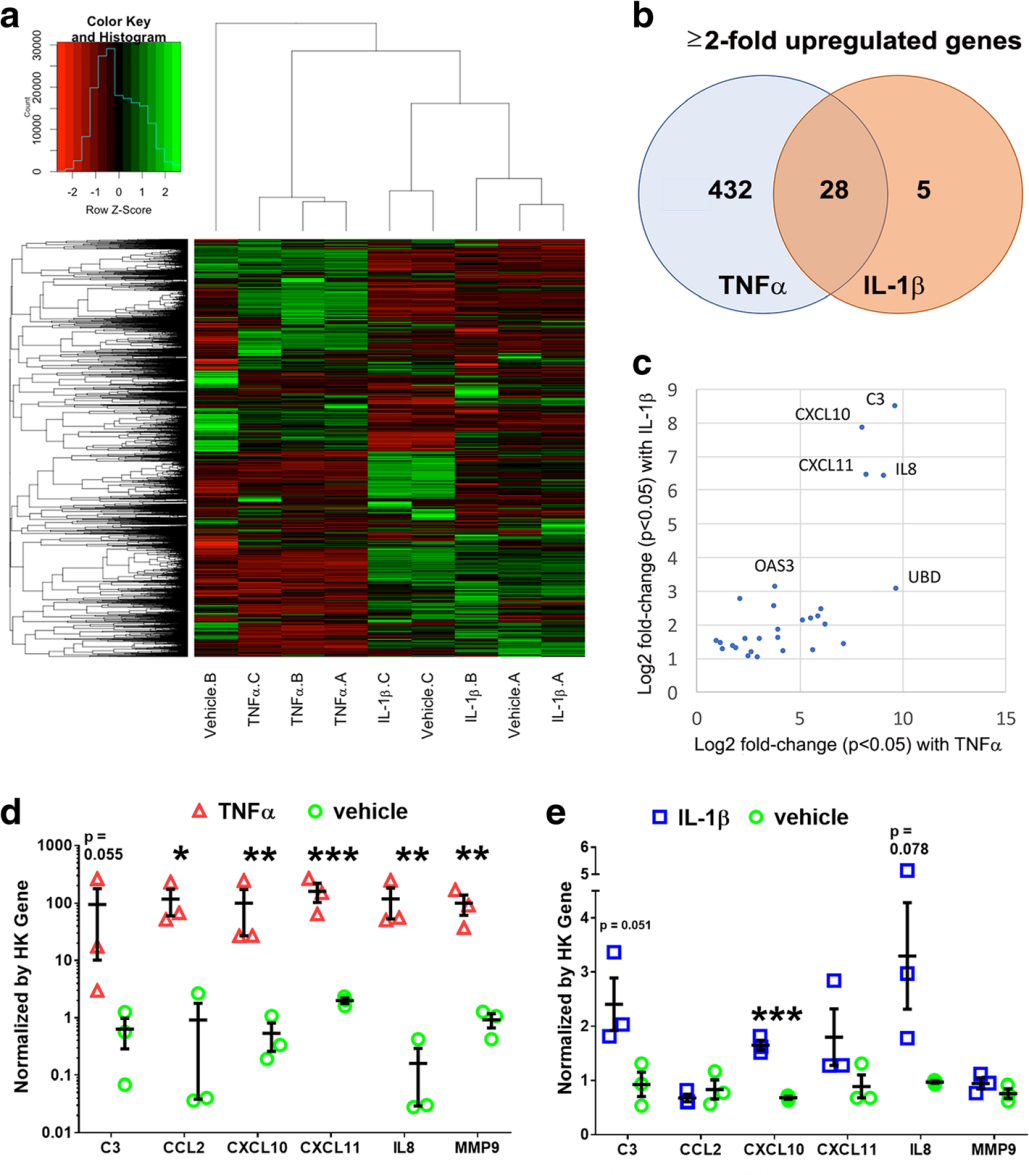
RNA sequencing reveals TNFα and IL-1β treatment result in the activation of neuroinflammatory genes with different effect sizes. **a** Unsupervised hierarchical clustering of the whole transcriptome of TNFα-, IL-1β-, and vehicle-treated differentiated astrocytes clusters TNFα samples by treatment. In contrast, the majority of IL-1β samples cluster more closely to the same passage vehicle control. **b** Venn diagram of ≥ + 2-fold change reveals differential gene activation effect sizes but overlapping upregulated genes by TNFα and IL-1β treatment. **c** Genes highly upregulated by either TNFα or IL-1β treatment have neuroinflammatory functions. **d** Independent verification of a cohort of six TNFα activated genes by qRT-PCR expression screening following 7 days TNFα treatment. **e** Independent qRT-PCR expression screening of the same six gene cohort following 7 days IL-1β treatment. Although IL-1β results in elevated mRNA levels for all six genes relative to control, the magnitude of increased expression is considerably reduced when compared to the TNFα qRT-PCR dataset (note log10 scale for TNFα compared to linear scale for IL-1β data). *** *p* < 0.001, **p < 0.01, *p < 0.05 unpaired Students *t* test, one-way ANOVA with Sidak’s multiple comparisons test *n* = 3. Data are presented as mean ± SD

Having established TNFα effects on NCRM-1 astrocyte function are consistent with a neuroinflammatory astroglia response, we next performed an unbiased whole-transcriptomics analysis of cytokine-treated astrocytes to further investigate neuroinflammatory gene signatures. NCRM-1 astrocytes were chronically treated with 100 ng/mL TNFα, IL-1β, or vehicle controls (*n* = 3). mRNA libraries were generated, clustered, and sequenced, and differential gene expression analysis was performed. Unsupervised hierarchical clustering revealed TNFα-treated astrocytes clustered separately, while IL-1β-treated samples clustered together with the vehicle-treated NCRM-1 passage control (Fig. 3a). Differential expression analysis of the 19,563 transcripts expressed in NCRM-1 astrocytes (Fragments Per Kilobase of transcript per Million mapped reads, FPKM > 1) resulted in the identification of 461 genes significantly deregulated with TNFα treatment compared to vehicle controls (*p* < 0.05 and log FC > 1). In contrast, IL-1β treatment resulted in only 33 significantly differentially expressed genes compared to vehicle controls, all of which are activated. Notably, a cross-comparison of genes significantly upregulated by IL-1β and TNFα treatments reveals a large overlap between IL-1β-activated and TNFα-activated gene datasets: almost all IL-1β-upregulated genes (28 out of 33) were also upregulated by TNFα (Fig. 3b). Genes highly upregulated by both TNFα and IL-1β treatment include the complement gene C3, chemokine genes CXCL10 and CXCL11, and the cytokine gene IL8 (Fig. 3c), consistent with both cytokines eliciting a neuroinflammatory response.

The greater number of differentially expressed genes with TNFα compared to IL-1β is consistent with a more widespread transcriptional response to this cytokine in differentiated astrocytes. Our whole-transcriptomics analysis therefore supports a more robust neuroinflammatory transcriptional response to TNFα compared to IL-1β. These results were independently validated by an orthogonal assay using qRT-PCR of a selected neuroinflammatory gene cohort. We confirmed that six neuroinflammatory genes (C3, CCL2, CXCL10, CXCL11, IL8, and MMP9) showed a greater gene upregulation by orders of magnitude in response to TNFα compared to IL-1β treatment (Fig. 3d, e). We next examined upregulated gene pathways by unbiased gene ontology (GO). GO searches reveal gene cohorts functioning in host defense to pathogens or injury, cytokine production, extracellular function, and the regulation of I-κβ kinase/NF-κβ cascade are activated by TNFα (Table 2). IL-1β similarly activates GO gene cohorts functioning in host defense to pathogens or injury as well as genes functioning in antigen presentation and leukocyte migration (Table 2). Taken together, the RNA sequencing data reveals a neuroinflammatory transcriptional response to TNFα and IL-1β in differentiated astrocytes. The number of differential expressed genes and the magnitude of neuroinflammatory gene activation changes are greater in response to TNFα compared to IL-1β treatment consistent with the greater effect of TNFα on NF-κβ signaling, glutamate uptake, and phagocytosis.

**Table 2:** Gene ontology (GO) analysis for all significantly upregulated genes by chronic treatment with TNFα (2122 genes) or Il-1β (156 genes) using DAVID. Listed is the highest representative GO annotation term by false discovery rate (FDR) for each of the four highest clusters ranked by cluster enrichment score. Also listed is the total number of genes for each annotation category, the percentage of TNFα or Il-1β upregulated genes within this annotation, the fold-enrichment of this GO gene set, and the FDR corrected *p* values for each GO enrichment

Genes upregulated with TNFα treatment						
Cluster	Cluster enrichment score	GO term	Total number of genes in category	% of TNFα upregulated genes in category	Fold enrichment	FDR
1	8.81	GO:0006952~defense response	118	5.98	1.75	1.77E−06
2	6.61	GO:0001817~regulation of cytokine production	50	2.54	2.52	2.02E−06
3	6.58	GO:0044421~extracellular region part	159	8.06	1.59	2.14E−06
4	5.57	GO:0043122~regulation of I-kappaB kinase/NF-kappaB cascade	32	1.62	2.73	4.43E−04
Genes Upregulated with IL-1β treatment						
Cluster	Cluster enrichment score	GO Term	Total number of genes in category	% of IL-1β upregulated genes in category	Fold enrichment	FDR
1	4.77	GO:0006952~defense response	25	17.36	4.70	5.451E−07
2	3.30	GO:0019882~antigen processing and presentation	11	7.64	15.32	3.451E−06
3	2.39	GO:0050900~leukocyte migration	7	4.86	4.20	0.01
4	2.19	GO:0070011~peptidase activity, acting on L-amino acid peptides	12	8.33	2.68	6.12

**Fig. 4:**
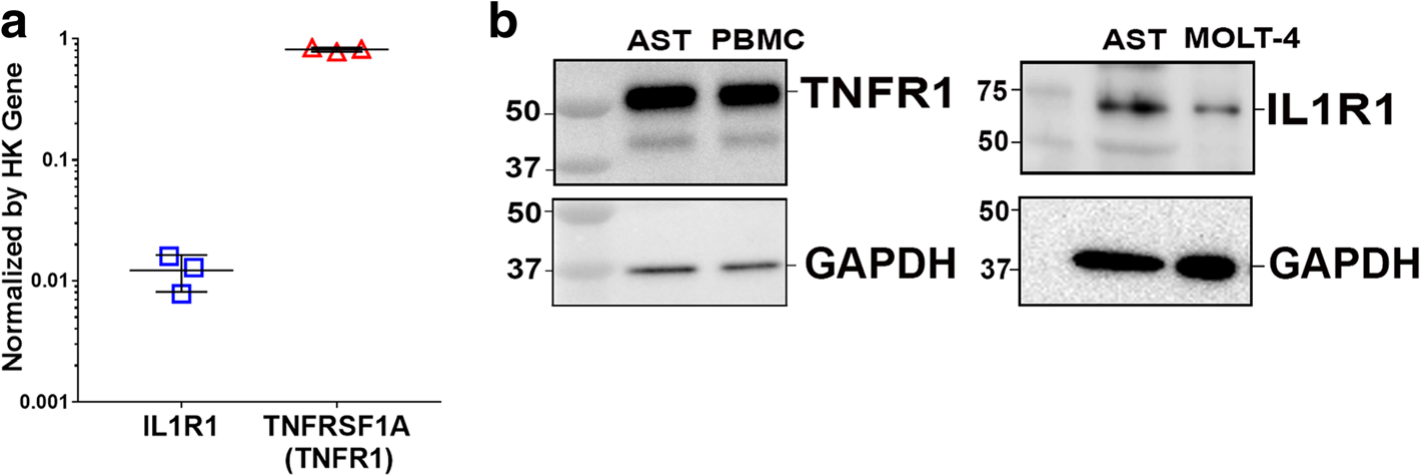
Expression analysis reveals comparatively higher TNFα compared to IL-1β receptor expression in differentiated astrocytes. **a** qRT-PCR confirms higher mRNA levels for canonical TNFα receptor gene TNFR1 (TNFRSF1A) compared to canonical IL-1β receptor IL1R1 in differentiated astrocytes. **b** Western blot confirms higher TNFR1 compared to IL1R1 protein expression in differentiated astrocytes (AST). Peripheral blood mononuclear cells (PBMC), and MOLT-4 cell lysates were included as positive TNFR1 and IL1R1 controls respectively

Next, we examined the RNA-seq data for a possible explanation for the differential effects of TNFα compared to IL-1β on differentiated astrocytes. Messenger RNA transcript levels (Fragments Per Kilobase of transcript per Million mapped reads) are consistent with greater expression of TNFα compared to IL-1β receptor-signaling genes in NCRM-1 astrocytes (not shown). Higher mRNA levels of the canonical TNFα receptor gene TNFR1 (gene name TNFRSF1A) compared to the canonical IL-1β receptor IL1R1 were confirmed by qRT-PCR (Fig. 4a), and Western blots using antibodies to the same canonical TNFα and IL-1β receptors reveal comparatively greater expression of TNFR1 compared to IL1R1 protein in NCRM-1 astrocytes (Fig. 4b). Thus, differences in the expression levels of canonical receptors for TNFα and IL-1β could account for the differential effects of these cytokines on differentiated NCRM-1 astrocytes.

## Discussion

The development of human stem cell culture models of neuroinflammatory astrocytes is expected to advance our understanding of the pathological roles of this cell type in CNS trauma, infection, and disease. In this study, we demonstrate human iPSC-derived NSPCs can be rapidly differentiated to neuroinflammatory reactive astrocytes. We show chronic TNFα treatment in culture activated NF-κβ signaling in differentiated astrocytes to depress glutamate uptake, increase phagocytic activity, and trigger widespread changes in gene expression that included the activation of classical pro-inflammatory gene targets. These data demonstrate our iPSC-derived astrocyte model provides an easily accessible human cell culture system for investigating TNFα reactive astroglia in CNS injury, infection, and disease.

Reactive astrogliosis in the CNS has recently been revealed to be a divergent stimulus-dependent cell state. Reactive astrocytes in lipopolysaccharide (LPS) injection models of systemic infection are neurotoxic (referred to as A1 astrocytes), whereas reactive astrocytes in a stroke model of middle cerebral artery occlusion (MCAO) are neuroprotective (referred to as A2 astrocytes) [13]. A1 reactive astrocytes are induced by reactive microglia, and screening identifies the candidate microglia proteins TNFα, IL-1α, and C1q as the strongest inducers of a partial A1 astrocyte phenotype [13]. LPS significantly increases the secretion of TNFα, IL-1α, and C1q by microglia in vitro, confirming these three microglia-derived proteins are key drivers of A1 astrocytic states. LPS-activated microglia also secrete IL-1β, but the cytokine is not able to induce A1 transcripts indicating a prominent TNFα but not IL-1β signaling component in A1 astrogliosis. Accordingly, we speculate chronic TNFα treatment induces a partial A1 astroglial fate in NCRM-1 astrocytes. Interestingly, it should be noted we observed increased general phagocytic activity with TNFα treatment in contrast to decreased synaptosome phagocytosis reported in A1 astrocytes [13]. It is feasible that the physiological removal of synaptosomes compared to the pathological removal of cell debris (modeled in our assay) are differentially affected by A1 induction consistent with this disparity in astrocyte phagocytosis data. However, this remains to be demonstrated.

In contrast to the robust response to TNFα, astrocyte responses to IL-1β were attenuated. Although chronic IL-1β treatment similarly elicited pro-inflammatory gene upregulation, the scope and magnitude of gene changes were substantially reduced when compared to TNFα-treated astrocytes. Furthermore, IL-1β did not significantly activate NF-κβ signaling or alter glutamate uptake or phagocytosis in our in vitro assays. We provide transcriptome data and Western blot evidence this attenuated response could be due to comparatively lower levels of the cognate receptor for IL-1β in our differentiated astrocytes.

NSPCs cultured under our astrocyte culture conditions rapidly develop CD44 antigen immunoreactivity within the first 1–3 weeks, assume a fibrous or protoplasmic-like morphology, and express multiple glial cell marker proteins after ≈ 35 days differentiation [17]. Despite the expression of astrocyte markers such as GFAP, S100β, and VIMENTIN, RNA-seq expression screening data of differentiated NCRM-1 astrocytes is more consistent with an immature astrocyte progenitor cell (APC) state than a mature astrocyte cell status [17]. It is therefore possible that an immature APC-like status of our differentiated astrocytes could account for the attenuated response to IL-1β. Another possibility is that cell culture artifacts are responsible. A recent 2017 study of inflammation-responsive human iPSC-derived astrocytes reported strong upregulation of pro-inflammatory genes in response to IL-1β [17]. In this study, astrocytes were differentiated using a neuronal medium with leukemia inhibitory factor (LIF) and serum. Our astrocytes were differentiated from iPSC-derived NSPCs in media containing the growth factor Heregulin and the TGF-beta superfamily member Activin A with serum [16]. Although there is signaling convergence between these two astrocyte protocols with both Heregulin and LIF signaling through JAK/STAT3 and PI3/AKT pathways and Activin enhancement of LIF-mediated astrocyte differentiation [35], it is highly likely the two protocols generate transcriptionally distinct astrocytes. Furthermore, the NSPCs differentiated to astrocytes in our study are positionally naïve, whereas the 2017 study used dorsal forebrain NSPCs following SMAD inhibition patterning. Thus, our cultures represent a highly rapid system for modeling A1-like TNFα-responsive astroglial states but may not be applicable to astrogliosis in all neuroinflammatory conditions or CNS regions.

In this study, we highlight the benefits of a readily accessible cell source, relatively simple technical procedures, and high reproducibility of iPSC-based approaches to model human astrocyte pathology in the laboratory. However, it should be noted that the differentiation of astrocytes from progenitors in cell culture has been linked to a reactive astroglia transcriptome in cultured astrocytes that may blunt or bias experimental outcomes [12]. In contrast, the transcriptome of astrocytes isolated by immuno-panning from surgically resected human brain or fetal tissue is similar to that of in vivo astrocytes and is considered a more physiological astrocyte state [1]. Although this suggests caution should be applied when evaluating iPSC-derived astrocyte studies, stem cell culture methods remain the more practical option for modeling neurological conditions in culture due to the problems of tissue availability for immuno-panning combined with the difficulty of replicating disease-related genomic conditions in immuno-isolated astrocytes.

## Conclusion

In conclusion, this manuscript describes a rapid protocol for in vitro modeling neuroinflammatory human astrocytes in CNS trauma or neurological diseases. Using this protocol, NCRM-1 astrocyte can be used for high-through screens to identify novel anti-inflammatory drugs to treat CNS trauma or disease.

## Data Availability

The datasets used and/or analyzed during the current study are available from the corresponding author on reasonable request and/or data will be deposited with the Genome Expression Omnibus https://www.ncbi.nlm.nih.gov/geo/

## Abbreviations

ADM: Astrocyte differentiation medium
CNS: Central nervous system
EB: Embryoid body
FC: Fold change
FPKM: Fragments Per Kilobase of transcript per Million mapped read
GO: Gene ontology
IL-1β: Interleukin-1 beta
iPSC: Induced pluripotent stem cell
LPS: Lipopolysaccharide
MCA: Middle cerebral artery
MFI: Mean fluorescence intensity
NSPC: Neural stem progenitor cell
TNFα: Tumor necrosis factor alpha
